# Beyond QRS Duration: Myocardial Work Indices for the Assessment of Left Bundle Branch Block

**DOI:** 10.3390/biomedicines14040941

**Published:** 2026-04-21

**Authors:** Magdalena Potapowicz-Krysztofiak, Martyna Dąbrowska, Małgorzata Maciorowska, Zbigniew Orski, Paweł Krzesiński, Marek Kiliszek, Beata Uziębło-Życzkowska

**Affiliations:** Department of Cardiology and Internal Diseases, Military Institute of Medicine—National Research Institute, 04-141 Warsaw, Poland; mdabrowska4@wim.mil.pl (M.D.); mmaciorowska@wim.mil.pl (M.M.); zorski@wim.mil.pl (Z.O.); pkrzesinski@wim.mil.pl (P.K.); mkiliszek@wim.mil.pl (M.K.); buzieblo-zyczkowska@wim.mil.pl (B.U.-Ż.)

**Keywords:** myocardial work, dyssynchrony, speckle tracking echocardiography, left bundle branch block, QRS duration, HFrEF

## Abstract

**Background:** Left bundle branch block (LBBB) and QRS prolongation are markers of electrical dyssynchrony in heart failure, but they do not fully reflect its mechanical consequences. Myocardial work (MW)-derived indices may provide a more comprehensive assessment of left ventricular (LV) mechanical dyssynchrony. We evaluated associations between LV MW parameters, QRS duration, and LBBB in patients with heart failure with reduced ejection fraction (HFrEF) referred for ICD/CRT implantation. **Methods:** In this single-centre observational cross-sectional study, 96 consecutive patients referred for ICD or CRT implantation were screened. All patients underwent standardized baseline comprehensive echocardiography followed by advanced MW analysis. Myocardial work index (MWI) dispersion was assessed using two complementary methods. MWI dispersion (SD) was calculated as the standard deviation of segmental MWI values across all LV segments, and MWI dispersion (IQR) was defined as the interquartile range (IQR) of segmental MWI values. We evaluated the associations between QRS duration and MW-derived dyssynchrony parameters (individual and composite), as well as their discriminative performance for LBBB. Seven patients were excluded from further analysis due to inadequate echocardiography image quality. **Results:** The final study group comprised 89 patients with HFrEF (median age 65.5 years), of whom 67.4% were assigned to CRT. LBBB was present in 41.6%, and the median QRS duration was 142 ms (112–162). All analyzed LV MW indices were significantly associated with QRS duration (all q < 0.01). The strongest correlations were observed for MWI dispersion (IQR) (r = 0.58), peak strain dispersion (PSD) (r = 0.54), lateral–septal work asymmetry (r = 0.53), and MWI dispersion (SD) (r = 0.52) (all q < 0.0001). All MW indices differed significantly between patients with and without LBBB (all q ≤ 0.0001). MWI dispersion (IQR) showed the best single-marker discrimination of LBBB (AUC = 0.852). Composite indices achieved AUC = 0.84 but did not significantly improve discrimination versus MWI dispersion (IQR) alone. **Conclusions:** Myocardial work-derived indices of left ventricular dyssynchrony are strongly associated with QRS duration and the presence of LBBB in patients with HFrEF. Among them, MWI dispersion (IQR) was shown to be the best-performing MW marker for identifying LBBB. These findings suggest that MW dispersion may serve as a robust echocardiographic marker of mechanical dyssynchrony and warrants further investigation as a potential tool for predicting CRT response.

## 1. Introduction

Left bundle branch block (LBBB) represents a major electrical conduction abnormality that profoundly alters left ventricular (LV) electromechanical coupling. It still constitutes an important basis for qualifying patients for cardiac resynchronization therapy (CRT) implantation [1]. Beyond its characteristic electrocardiographic (ECG) features, LBBB is associated with mechanical dyssynchrony and regional disparities in myocardial loading, ultimately contributing to adverse ventricular remodeling and impaired cardiac function [2]. The large meta-analysis conducted by Martins et al. [3] showed that LBBB morphology was the most powerful independent predictor of echocardiographic response to CRT. Patients presenting with LBBB achieved significantly greater rates of LV reverse remodeling than those without LBBB, underscoring the pivotal role of LBBB in optimizing patient selection for CRT. Although QRS duration remains the cornerstone for the ECG diagnosis and therapeutic decision-making in LBBB, it provides only an indirect and incomplete reflection of the underlying mechanical consequences.

Conventional echocardiographic parameters, including left ventricular ejection fraction (LVEF) and global longitudinal strain (GLS), capture global systolic function but are limited in their ability to characterize regional mechanical heterogeneity and work inefficiency induced by conduction disturbances. Advanced deformation imaging has improved the assessment of mechanical dyssynchrony [4,5]. However, strain-based parameters alone do not account for afterload, limiting their physiological interpretability, particularly in patients with abnormal ventricular activation patterns.

Non-invasive myocardial work (MW) analysis, derived from pressure–strain loops, integrates myocardial deformation with estimated LV pressure, offering a load-adjusted assessment of both global and regional LV function [6,7,8]. This approach enables the quantification of global constructive and wasted work (GCW and GWW), as well as global work efficiency (GWE), providing insights into the energetic consequences of electromechanical dyssynchrony. Importantly, LBBB is characterized by pronounced regional disparities in myocardial work, suggesting that measures of MW dispersion may represent a sensitive marker of its mechanical signature.

Recent studies have highlighted the potential role of mechanical dispersion and phase-based indices, such as peak strain dispersion (PSD), in the assessment of ventricular dyssynchrony [9,10]. However, data on comprehensive MW dispersion and its integration into composite indices remain limited. Moreover, it is unclear whether such composite MW-based measures provide incremental information beyond QRS duration in identifying LBBB-related mechanical dysfunction.

Therefore, the present study in patients with heart failure with reduced ejection fraction (HFrEF) aimed to systematically evaluate left ventricular myocardial work (LV MW) parameters and their dispersion, quantified using multiple complementary statistical approaches, and to construct composite indices based on the strongest associations with QRS duration and the presence of LBBB. In addition, we assessed the ability of these indices to discriminate patients with LBBB and to determine whether they provide incremental information beyond standard ECG measures.

## 2. Methods

### 2.1. Study Population

A prospective study enrolled consecutive adult patients in sinus rhythm, with no history of atrial fibrillation, who were diagnosed with HFrEF (LVEF ≤ 40%) regardless of the aetiology of HF, and were referred for ICD/CRT implantation. Patients were qualified for the procedure in accordance with current guideline recommendations [1]. Exclusion criteria included: severe Chronic Obstructive Pulmonary Disease (stage C or D), hemodynamically significant aortic or mitral stenosis, hypertrophic cardiomyopathy, and recent (<3 months) acute coronary syndrome or coronary revascularization. In all patients, an ECG and a comprehensive transthoracic echocardiographic (TTE) examination were performed on the day preceding the procedure. For the diagnosis of LBBB on ECG, the criteria proposed by Strauss et al. were applied [11]. Complete LBBB was defined as a QRS duration ≥140 ms in men or ≥130 ms in women, together with mid-QRS notching or slurring in at least two contiguous leads (I, aVL, V1, V2, V5, or V6).

### 2.2. Echocardiographic Assessment and Myocardial Work Analysis

All patients underwent a comprehensive TTE performed according to current recommendations of the European Association of Cardiovascular Imaging [12,13]. Standard two-dimensional, Doppler, and tissue Doppler images were acquired using commercially available ultrasound systems (VividE95, GE Healthcare, Horten, Norway). Left ventricular dimensions, volumes, and ejection fraction were assessed using the biplane Simpson method.

Left ventricular myocardial work was assessed using a non-invasive pressure–strain loop methodology integrating speckle tracking-derived longitudinal strain with estimated LV pressure curves adjusted to brachial systolic blood pressure [6]. Global and regional myocardial work parameters were calculated, including global work index (GWI), GCW, GWW, and GWE. In addition, segmental myocardial work indices were derived for all 17 LV segments.

**Figure 1:**
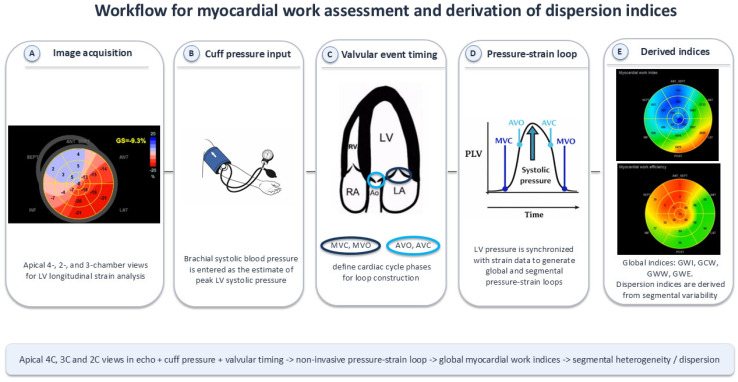
Schematic illustration of myocardial work assessment. Speckle-tracking echocardiography and brachial cuff systolic blood pressure were used to construct a non-invasive left ventricular pressure–strain loop. On this basis, global and segmental myocardial work parameters were calculated, including global constructive work, global wasted work, and global work efficiency. Dispersion-based indices were subsequently derived from the variability of regional myocardial work values across all left ventricular segments. Abbreviations: AVO, aortic valve opening; AVC, aortic valve closure; GCW, global constructive work; GWE, global work efficiency; GWI, global work index; GWW, global wasted work; LV, left ventricular; MVC, mitral valve closure; MVO, mitral valve opening; MW, myocardial work.

For the assessment of mechanical dyssynchrony, novel echocardiographic parameters derived from MW analysis were selected, as they capture three complementary aspects of LV dyssynchrony: local, temporal, and global. Local dyssynchrony was evaluated using indices of regional MW asymmetry between LV segments: L-S Work Asymmetry as a difference in myocardial work index (MWI) between the basal lateral and basal septal LV segments, and P-AS Work Asymmetry as a difference in MWI between the basal posterior and basal anteroseptal LV segments. Temporal dyssynchrony was assessed using peak strain dispersion (PSD), defined as the standard deviation of time to peak systolic strain across LV segments. Global dyssynchrony and mechanical inefficiency were assessed using global myocardial work indices, including dispersion- and range-based measures. This approach enables a comprehensive evaluation of the mechanical consequences of electrical dyssynchrony observed on ECG. Figure 1 presents a schematic overview of the myocardial work assessment workflow, including image acquisition, construction of the LV pressure–strain loop, and derivation of myocardial work efficiency-related parameters.

Mechanical dispersion of myocardial work was comprehensively evaluated using three complementary statistical approaches. Myocardial work dispersion indices were calculated separately for myocardial work index (MWI) and myocardial work efficiency (MWE) as:

1. the standard deviation (SD) of segmental values
2. the Interquartile Range (IQR), and
3. the range (maximum–minimum difference) across all LV segments.

This multi-metric approach allowed for a robust characterization of heterogeneity in regional MW distribution. All myocardial work parameters and dispersion indices were subsequently included in correlation and predictive analyses with ECG variables, including QRS duration and the presence of LBBB.

### 2.3. Ethical Statement

All study procedures were conducted in accordance with the principles of Good Clinical Practice and the Declaration of Helsinki. The study protocol was approved on 16 October 2024 by the local institutional ethics committee (Resolution No. 52/2024). Written informed consent was obtained from all participants before inclusion in the study.

### 2.4. Statistical Methods

Statistical analyses were performed in the R environment (version 4.5.2; R Foundation for Statistical Computing, Vienna, Austria). A *p* < 0.05 was considered statistically significant.

Continuous variables were tested for normality with the Shapiro–Wilk test and are presented as mean ± SD or median (IQR), as appropriate. Categorical variables are presented as counts and percentages. Between-group comparisons (LBBB vs. non-LBBB) were performed using the Mann–Whitney U test for continuous variables and the χ^2^ test or Fisher’s exact test for categorical variables.

The relationship between QRS and individual indicators was assessed using Spearman’s rank correlation coefficient (rho). Differences in indicator values between groups with and without LBBB were assessed using the Mann–Whitney test. Results are presented as median and interquartile range (IQR). In addition, the area under the ROC curve (AUC) was calculated as a measure of the discriminatory ability of the indicators in identifying LBBB. A correction for multiple comparisons was applied using the FDR (Benjamini–Hochberg) method.

Next, the total rank (sum of ranks) was calculated, and the indicators with the lowest total rank were selected for the construction of the index. The index was calculated as the standardized sum (mean of z-scores) of selected indicators. ROC curves and AUC with 95% confidence intervals were calculated for the selected indices.

Linear regression was performed to assess the independent relationship between QRS and mechanical indices. Models including selected indices and models containing single indices were analyzed. The predictive quality of the models was compared based on the mean square error (MSE). MSE differences between model pairs were assessed using the *t*-test for prediction error differences.

Logistic regression was used to identify independent predictors of LBBB. The discriminatory power of the logistic models was compared using the DeLong test for dependent ROC curves [14]. In addition, the fit of the models was assessed based on the AIC and BIC information criteria.

To assess intra-observer reproducibility, repeated measurements were performed in 20 randomly selected patients. The same observer repeated the offline myocardial work analysis after an interval of several days and was blinded to the results of the initial assessment. Intra-observer agreement was assessed for conventional myocardial work parameters and for segmental work values. Reproducibility was evaluated using the intraclass correlation coefficient (ICC), together with Bland–Altman analysis and coefficient of variation.

## 3. Results

A total of 96 patients referred for ICD/CRT implantation were initially screened. After exclusion of seven patients due to inadequate echocardiographic image quality, 89 patients were ultimately included in the analysis. Among the study population, 60 (67.4%) patients were assigned to receive a CRT device.

The median age of the study population was 65.5 years (56–74), and the majority were men (78 patients, 87.6%). The median BMI was 26.5 kg/m^2^ (24.5–29). All patients met the diagnostic criteria for HFrEF, with a median LVEF of 32% (26–35). Ischemic aetiology of HF was present in 61 (68.5%) patients. ECG and echocardiographic examinations were performed at a median heart rate of 67 beats per minute (IQR 60–80). LBBB was present in 37 (41.6%) patients. The median QRS duration in the entire cohort was 142 ms (112–162).

A comparison of patients with and without LBBB in terms of basic clinical, ECG, and echocardiographic parameters is presented in Table 1.

### 3.1. Associations Between QRS Duration and Left Ventricular Myocardial Work Indices

All analysed indices of LV MW showed significant correlations with QRS duration after False Discovery Rate (FDR) correction (Table 2, all *p* values < 0.01 and 0.02 for GWI). Among the top-ranked parameters, the strongest positive correlations were observed for parameters reflecting mechanical dyssynchrony and heterogeneity of myocardial work, including MWI dispersion (IQR) (r = 0.58, q < 0.0001), PSD (r = 0.54, q < 0.0001), L–S work asymmetry (r = 0.53, q < 0.0001), and MWI dispersion (SD) (r = 0.52, q < 0.0001). As expected, GWE and GWI correlated negatively with QRS duration (r = −0.46 and −0.25, respectively), whereas GWW showed a weaker positive correlation (r = 0.28, q = 0.0081).

In linear regression analyses, the highest-ranked individual LV MW parameters (MWI dispersion [IQR], L–S work asymmetry, and PSD) significantly predicted QRS duration (β = 0.053, SE = 0.008, *p* < 0.00001; β = 0.019, SE = 0.004, *p* < 0.00001; and β = 0.341, SE = 0.061, *p* < 0.00001, respectively).

### 3.2. Comparison of LVMW Between LBBB and Non-LBBB Patients

Almost all LV MW indices differed significantly between patients with and without LBBB (Table 3, all FDR-adjusted q ≤ 0.0001). Patients with LBBB demonstrated markedly higher values of MW dispersion, work asymmetry, PSD, and GWW, along with lower GWE.

In ROC analyses, the strongest single-maker discrimination of LBBB was observed for MWI dispersion (IQR) (AUC = 0.852), followed by MWI dispersion (SD) (AUC = 0.821) and L–S work asymmetry (AUC = 0.807). PSD showed slightly lower discrimination (AUC = 0.775). Operating characteristics differed by variable: MWI dispersion (IQR) and L–S work asymmetry supported high-specificity thresholds, while PSD favoured higher sensitivity at the expense of specificity. Univariable logistic regression confirmed that each LV MW measure was significantly associated with LBBB status (all *p* ≤ 0.0003).

To prioritize variables for subsequent modelling, LV MW indices were ranked according to a combined assessment of correlation strength with QRS duration (Spearman rho rank) and discrimination of LBBB (AUC rank), summarized as a composite ranking score (Table S1). In this ranking, the highest-priority variables were MWI dispersion (IQR), L–S work asymmetry, MWI dispersion (SD), and PSD. Because MWI dispersion (IQR) and MWI dispersion (SD) represent closely related dispersion-derived parameters and were strongly intercorrelated, only one was retained to reduce redundancy in further model construction. MWI dispersion (IQR) was selected as the representative dispersion parameter (higher overall rank and best single-marker discrimination), and therefore the subsequent composite models were built using MWI dispersion (IQR), L-S Work Asymmetry, and PSD.

### 3.3. Composite MW Dyssynchrony Indices and Model Comparisons

To summarize dyssynchrony across complementary fields (dispersion, asymmetry, and timing), composite MW dyssynchrony indices were constructed using Z-score combinations of the highest-ranked variables. The first composite index consisted of two components: MWI dispersion (IQR) and L-S Work Asymmetry (Model 1), whereas the second one represented an extension of the first by the addition of PSD (Model 2). The composite indices showed strong associations with QRS duration and demonstrated very good discrimination of LBBB (AUC = 0.84 for both models), reflecting the benefit of integrating multiple factors of mechanical dyssynchrony into a single score. However, DeLong tests did not demonstrate a statistically significant AUC improvement of either composite index over the best single marker MWI dispersion (IQR) (Model 1 vs. MWI dispersion [IQR]: *p* = 0.721; Model 2 vs. MWI dispersion [IQR]: *p* = 0.916). Likewise, comparisons with L-S asymmetry showed only non-significant trends (*p* = 0.088 and *p* = 0.0748, respectively for both models). In contrast, Model 2 showed a statistically significant AUC improvement over PSD alone (*p* = 0.040).

Notably, information criteria supported the composite approach despite similar AUC values in some comparisons. In particular, both composite logistic models showed lower AIC/BIC than corresponding single-marker models based on L-S asymmetry or PSD, and Model 2 also showed lower AIC/BIC than MWI dispersion (IQR), whereas for Model 1 versus MWI dispersion (IQR), the AUC slightly favoured MWI dispersion (IQR) but AIC/BIC favoured the composite model. Detailed results of the comparison of logistic model discrimination using the DeLong test are presented in Table 4.

**Figure 2:**
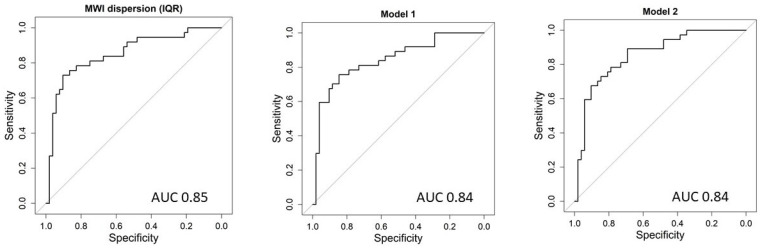
ROC analysis of the two constructed models and the best single parameter, MWI dispersion (IQR).

**Figure 3:**
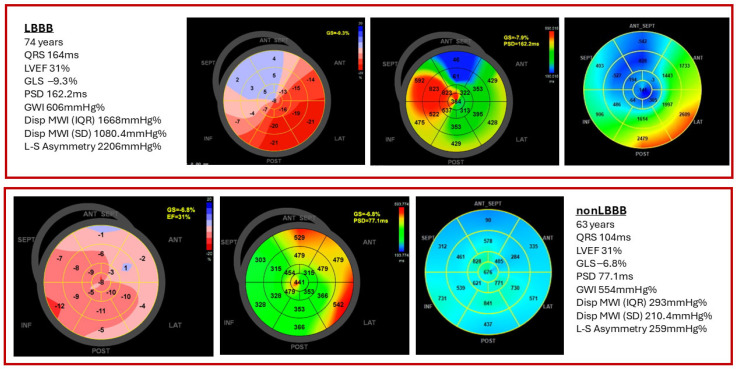
Representative echocardiographic images illustrating left ventricular longitudinal strain parameters and myocardial work indices in patients with and without left bundle branch block (LBBB). Abbreviations: LVEF, left ventricular ejection fraction; GLS, global longitudinal strain; PSD, peak strain dispersion; GWI, global work index; Disp MWI (IQR), dispersion of myocardial work index expressed as interquartile range; Disp MWI (SD), dispersion of myocardial work index expressed as standard deviation; L-S asymmetry, latero-septal wall asymmetry.

Figure 2 shows the ROC analysis for both constructed models and the best single parameter, MWI dispersion (IQR). Figure 3 shows representative echocardiographic images illustrating left ventricular longitudinal strain parameters and myocardial work indices in patients with and without LBBB. Collectively, these findings identify MWI dispersion (IQR) as the best-performing single MW marker for LBBB discrimination, while composite dyssynchrony indices provide a robust summary phenotype with improved global model fit and significantly better discrimination than PSD.

#### Intra-Observer Variability

Intra-observer reproducibility was good to excellent across all analyzed MW indices. ICC values ranged from 0.931 to 0.991 for global myocardial work parameters and from 0.967 to 0.979 for segmental myocardial work indices. Coefficients of variation were low, ranging from 2.0% to 9.6%, and Bland–Altman analysis demonstrated small mean differences between repeated measurements.

## 4. Discussion

In our study cohort, myocardial work-derived dyssynchrony measures were consistently linked to electrical conduction delay and the presence of LBBB. Across the top-ranked parameters, longer QRS duration was associated with greater mechanical dispersion and dyssynchrony, including higher MWI dispersion (both IQR and SD), greater L-S work asymmetry, and larger peak-systolic dispersion. Our findings support the concept that a wider QRS complex reflects not only delayed electrical activation, but also more impaired mechanical function, characterized by heterogeneous regional work distribution and greater temporal dispersion of contraction [15,16].

Moreover, the principal finding of our study was that LBBB was significantly associated with greater MW dyssynchrony. Among individual markers, MWI dispersion (IQR) showed the strongest overall discrimination of LBBB and also behaved as the most informative single predictor in regression models, providing incremental information beyond PSD and L-S work asymmetry. This suggests that MW dispersion may capture an important feature of LBBB: different LV regions work unevenly, and this is closely linked to conduction abnormalities. In contrast, PSD, although sensitive, appeared to be less specific. This is consistent with the fact that PSD reflects temporal delays that can occur in many settings (e.g., variable loading conditions or regional dysfunction), and not only in classical LBBB-related dyssynchrony.

To date, the relationship between MWI dispersion and QRS duration or LBBB has not been investigated in a population similar to ours. In the STANISLAS population-based cohort [17], the authors evaluated electro-mechanical coupling in an initially healthy, community-based population (French-origin participants, 20–75 years old) without overt cardiac disease. Mechanical dyssynchrony was assessed by 2D speckle-tracking echocardiography using strain-derived temporal indices, mainly time-to-peak longitudinal strain (from QRS onset to peak strain across LV segments) and mechanical dispersion, defined as the standard deviation of time-to-peak strain across the 17 LV segments. In contrast to our findings, they did not observe a significant association between QRS duration and mechanical dispersion or time-to-peak strain. This discrepancy is likely explained by differences in study populations and the limited QRS variability in the STANISLAS cohort (mean QRS ~91 ms), which did not include patients with QRS >120 ms.

The review paper by Strik et al. [18] summarizes the knowledge on electrical and mechanical ventricular activation in LBBB and during CRT, with a focus on why some heart failure patients respond to CRT, while others do not. The authors discuss the pathophysiology of LBBB, including heterogeneous patterns of LV activation, and explain how these differences may influence CRT effectiveness. A key message is that “true” LBBB [19] represents an important electrical substrate for CRT response, but QRS duration alone is not sufficient to fully characterize the conduction abnormality or predict outcomes. The review also shows that echocardiographic dyssynchrony parameters (especially older timing-based indices) have had inconsistent performance in predicting CRT response, while newer approaches (e.g., speckle-tracking-based measures such as septal rebound stretch) appear more promising. The study by Gao et al. [20] supports the use of myocardial work-based assessment in mechanical dyssynchrony by showing that, even in patients with idiopathic LBBB and preserved LVEF, pressure–strain loop-derived indices detected clear abnormalities (lower GWI and GWE, higher GWW) together with regionally reduced MWI, particularly in the septal and apical segments. This suggests that myocardial work parameters are sensitive to electromechanical disturbance even when conventional systolic function (LVEF) remains preserved. In line with this concept, our results further extend the role of myocardial work in dyssynchrony assessment by showing that MW dispersion (based on 17-segment LV MWI heterogeneity) is strongly related to the presence of LBBB, supporting its value as a quantitative marker of LBBB-related mechanical discoordination.

Electrical markers such as QRS duration and LBBB morphology reflect abnormal ventricular activation and remain the basis for CRT qualification. However, they describe the timing and pattern of electrical conduction rather than its actual mechanical consequences. In contrast, MW-derived indices reflect the downstream mechanical expression of this delay, including regional work imbalance, temporal discoordination, and mechanical inefficiency. Therefore, electrical and MW-based parameters should not be viewed as competing markers, but rather as complementary descriptors of different aspects of dyssynchrony. This distinction may have clinical implications, as patients with similar electrical delay may still differ in the severity of mechanical discoordination. In this context, our study adds to this discussion by applying newer myocardial work-based markers of mechanical dyssynchrony rather than conventional time-based strain indices alone. We demonstrated that these MW dyssynchrony parameters were significantly associated not only with QRS duration, but also with the presence of LBBB. Importantly, they showed better performance than PSD, which is based mainly on temporal differences in activation/contraction across LV segments. This suggests that MW-based indices may provide a more comprehensive assessment of mechanical dyssynchrony, as they capture not only timing abnormalities but also heterogeneity in regional contractile work. As an adjunct to ECG-based assessment of electrical dyssynchrony, they may therefore represent promising echocardiographic markers for a more refined characterization of electromechanical discoordination.

Our proposed assessment of mechanical dyssynchrony using MWI dispersion (IQR) may not be the simplest echocardiographic measurement to obtain. However, with current technology, its measurement does not appear to be particularly complex. Moreover, as we showed in our study, this single parameter was sufficient to assess both QRS duration and the presence of LBBB. The composite Model 2 achieved the numerically highest AUC among evaluated parameters and improved discrimination relative to PSD alone. Importantly, however, AUC-based superiority over MWI dispersion (IQR) was not confirmed by DeLong testing. In our analysis, LR tests and AIC/BIC suggested that the composite index fit the data better, even though its AUC was similar. In practical terms, the composite index may improve risk estimation, but not enough to clearly improve patient ranking compared with the best single marker.

From a mechanistic point of view, the results were consistent across analyses. MWI dispersion (IQR) reflects differences in regional LV work, PSD reflects delays in contraction timing, and lateral–septal work asymmetry reflects imbalance between LV regions. The models showed that MWI dispersion (IQR) and PSD provide partly independent information about QRS duration, because both remained significant in linear analyses. This suggests that electrical conduction delay may lead to mechanical dyssynchrony through more than one pathway: delayed contraction timing and uneven regional work distribution. Therefore, the composite index may be a useful global marker of dyssynchrony, as it combines complementary features into a single parameter and may simplify clinical research reporting.

Clinically, the strong association of MW-based dyssynchrony parameters with LBBB and QRS duration supports their validity as markers of dyssynchrony. At the same time, the lack of a statistically significant AUC improvement with complex models (Model 1 and 2) over MWI dispersion (IQR) suggests that, for simple binary classification of LBBB, one good dispersion parameter may be enough. The composite score may still be useful in other clinical settings, especially when a broader description of the phenotype is needed. This may include prediction of CRT response, risk stratification in patients with borderline QRS duration, or longitudinal follow-up. In such settings, combining information from different dyssynchrony domains may be more important than improving AUC alone.

Importantly, MW-derived indices should not be considered diagnostic tools for LBBB in place of ECG. Their value lies in quantifying the mechanical expression of LBBB, including regional work heterogeneity and mechanical discoordination. This may help to better characterize the electromechanical substrate in patients with conduction abnormalities and support future studies on CRT response prediction. At present, MWI dispersion (IQR) should be regarded as neither a substitute for guideline-based ECG criteria for CRT eligibility nor a standalone screening tool, but rather as a complementary echocardiographic parameter that refines mechanical phenotyping in patients with similar ECG features.

### Strengths and Limitations

This study has several strengths. We analyzed a well-characterized HFrEF cohort referred for ICD/CRT implantation, with standardized baseline ECG and echocardiographic assessment. We examined multiple MW-based dyssynchrony parameters across complementary fields (dispersion, asymmetry, and timing) using a comprehensive statistical approach. Importantly, our findings identify MWI dispersion, especially MWI dispersion (IQR), as a strong marker of electrical–mechanical discoordination and provide a rationale for further studies assessing its value in predicting response to cardiac resynchronization therapy.

Several limitations should be acknowledged. This was a single-centre study with a moderate sample size. The observational cross-sectional design precludes causal inference and does not allow for assessment of prognostic value or direct prediction of CRT response. In addition, intercorrelations among MW dyssynchrony parameters may limit incremental value in multivariable or composite models. The proposed composite indices were derived in a data-driven manner and were not internally validated. Therefore, some degree of overfitting cannot be excluded, and their performance should be considered exploratory and hypothesis-generating until confirmed in independent cohorts. Another limitation of this study is that inter-observer variability was not assessed. Therefore, although intra-observer reproducibility was good, the reproducibility of myocardial work measurements and derived dispersion indices between different observers remains to be determined. Finally, statistical significance does not necessarily translate into clinical usefulness, which may depend on the intended application.

## 5. Conclusions

In summary, LV mechanical dyssynchrony and uneven myocardial work were closely related to QRS prolongation and the presence of LBBB in patients with HFrEF. MW dispersion and asymmetry parameters were strongly associated with conduction abnormalities and clearly differentiated patients with and without LBBB. GWI dispersion was the best single marker, while Model 2 provided a compact multidomain measure that performed better than PSD and showed better overall model fit, although it did not significantly improve AUC compared with MWI dispersion alone. These findings support the use of MW-based dispersion and dyssynchrony measures as a quantitative way to describe electrical–mechanical coupling in conduction abnormalities. These findings also suggest that MW dispersion warrants further investigation as a potential tool for refining CRT candidate selection and predicting CRT response.

## Figures and Tables

**Table 1:** A comparison of patients with and without LBBB in terms of basic clinical, ECG, and echocardiographic parameters.

	LBBB Yes (*n* = 37) Median (IQR)	LBBB No (*n* = 52) Median (IQR)	*p*-Value
Age [years] median (IQR)	66 (58.5–77)	64 (52.5–71)	0.059
BMI [kg/m^2^] median (IQR)	25.4 (24–28.4)	27.2 (24.9–29.1)	0.076
Gender [Female] n (%)	8 (21.6)	7 (13.5)	0.40
Echocardiographic parameters; median (IQR)			
RVDd [mm]	31 (29–33)	32 (30–35)	0.168
LVDd [mm]	64 (62–68.6)	63.8 (59–70)	0.425
LVEDV [ml]	189.5 (157.5–227.5)	176 (142.5–214)	0.164
LVSV [ml]	54 (42–65)	54.5 (47–64)	0.763
GCW [mmHg%]	1035 (866–1283.5)	1015 (824.5–1306)	0.582
LA area [cm^2^]	24.7 (21.5–27.8)	26.1 (21.9–31.5)	0.246
LAVI [ml/m^2^]	43.1 (38.8–51.4)	44.2 (36.3–58.9)	0.984
LV GLS [%]	7.7 (6.6–9.5)	8.1 (6–9.7)	0.667
LV EF [%]	28 (24–33)	33 (27.5–36.5)	0.007
TAPSE [mm]	19.6 (17–23)	19 (15–22)	0.519
S’ RV [cm/s]	10 (8–12)	10 (8–11)	0.724
e’avg [cm/s]	4 (3.5–5)	4.5 (3.5–5.5)	0.335
E/e’ avg	12.4 (10–17.1)	12 (8.6–17.3)	0.555
ECG parameters; median (IQR)			
Heart rate [beats/min]	71 (61–79)	66 (60–80)	0.744
P wave [ms]	118 (100–128)	116 (100–128)	0.867
PQ interval [ms]	180 (164–196)	173 (162–200)	0.771
QRS duration [ms]	160 (148–172)	118 (101–148)	<0.0001
Clinical data; *n* (%)			
Hypertension	25 (67.6)	34 (65.4)	0.83
Diabetes mellitus	30 (81.1)	40 (76.9)	0.99
Stroke	3 (8.1)	6 (11.5)	0.73
Myocardial infarction	19 (51.4)	37 (71.2)	0.08
Coronary artery disease	23 (62.2)	41 (78.8)	0.11
Hyperlipidemia	29 (78.4)	34 (65.4)	0.15
Chronic kidney disease	7 (18.9)	14 (26.9)	0.63
Smoking	16 (43.2)	28 (53.8)	0.29

Abbreviations: BMI, body mass index; E/e’ avg, average E to e’ ratio; GCW, global constructive work; IQR, interquartile range; LA, left atrium; LAVI, left atrial volume index; LVDd, left ventricular end diastolic diameter; LVEDV, left ventricular end-diastolic volume; LVEF, left ventricular ejection fraction; LV GLS, left ventricle global longitudinal strain; LV SV, left ventricular systolic volume; RVDd, right ventricular end diastolic diameter; S’ RV, right ventricular systolic velocity; TAPSE, tricuspid annular plane systolic excursion.

**Table 2:** Results of correlations between MW-derived LV dyssynchrony parameters and QRS duration.

	r Spearman	*p*-Value	q-Value (FDR-Adjusted)
GWW [mmHg%]	0.28	0.0081	0.0081
GWI [mmHg%]	−0.25	0.0209	0.0228
GWE [%]	−0.46	<0.0001	<0.0001
L-S Work Asymmetry [mmHg%]	0.53	<0.0001	<0.0001
P-AS Work Asymmetry [mmHg%]	0.45	<0.0001	<0.0001
MWI dispersion (SD) [mmHg%]	0.52	<0.0001	<0.0001
MWI dispersion (IQR) [mmHg%]	0.58	<0.0001	<0.0001
MWI dispersion (Range) [mmHg%]	0.47	<0.0001	<0.0001
MWE dispersion (SD) [mmHg%]	0.51	<0.0001	<0.0001
MWE dispersion (IQR) [mmHg%]	0.44	<0.0001	<0.0001
MWE dispersion (Range) [mmHg%]	0.45	<0.0001	<0.0001
PSD [ms]	0.54	<0.0001	<0.0001

Abbreviations: GWE, global work efficiency; GWI, global work index; GWW, global wasted work; IQR, interquartile range; L-S Work Asymmetry, difference in Global Work Index between the basal lateral and basal septal left ventricular segments; MWE, myocardial work efficiency; MWI, myocardial work index; P-AS Work Asymmetry, difference in Global Work Index between the basal posterior and basal anteroseptal left ventricular segments; PSD, peak strain dispersion; SD, standard deviation.

**Table 3:** Comparison of MW-derived LV dyssynchrony parameters between patients with and without LBBB (Mann–Whitney U test), with ROC-derived discriminative performance.

	LBBB No (*n* = 52) Median (IQR)	LBBB Yes (*n* = 37) Median (IQR)	*p*-Value	AUC	CI Low	CI High	*p*-Value
GWW [mmHg%]	268.5 (193.5–372.5)	433 (288–521)	0.0001	0.740	0.638	0.843	<0.0001
GWE [%]	79 (73–85)	71 (65–76)	0.0001	0.248	0.651	0.852	<0.0001
GWI [mmHg%]	736 (551–957.5)	682 (546–825)	0.4870	0.456	0.422	0.665	0.4802
L-S Work Asymmetry [mmHg%]	354.5 (22.5–922.5)	1425 (894–1708)	<0.0001	0.807	0.710	0.903	<0.0001
P-AS Work Asymmetry [mmHg%]	605.5 (83–972.5)	1418 (919–1979)	<0.0001	0.775	0.674	0.876	<0.0001
MWI dispersion (IQR) [mmHg%]	398 (333.5–545.5)	781 (614–976)	<0.0001	0.852	0.767	0.936	<0.0001
MWI dispersion (SD) [mmHg%]	354.2 (279.6–442.6)	627.9 (437.2–778.9)	<0.0001	0.821	0.731	0.911	<0.0001
MWI dispersion (Range) [mmHg%]	1246 (984.5–1723.5)	2222 (1546–2679)	<0.0001	0.800	0.709	0.892	<0.0001
MWE dispersion (SD) [mmHg%]	16.18 (12.6–19.8)	23.04 (19.4–26.2)	<0.0001	0.802	0.706	0.899	<0.0001
MWE dispersion (IQR) [mmHg%]	20 (15–26)	30 (20–37)	0.0008	0.709	0.596	0.822	0.0003
MWE dispersion (Range) [mmHg%]	57.5 (42.5–65)	75 (63–85)	<0.0001	0.780	0.683	0.877	<0.0001
PSD [ms]	84.25 (69.2–119.5)	140.1 (101.6–160.2)	<0.0001	0.775	0.679	0.871	<0.0001

Abbreviations: AUC—Area Under the Receiver Operating Characteristic Curve; CI low and CI high—the lower and upper 95% confidence interval for the AUC; GWE, global work efficiency; GWI, global work index; GWW, global wasted work; IQR, interquartile range; LBBB, left bundle branch block; L-S Work Asymmetry, difference in Global Work Index between the basal septal and basal lateral left ventricular segments; MWE, myocardial work efficiency; MWI, myocardial work index; P-AS Work Asymmetry, difference in Global Work Index between the basal posterior and basal anteroseptal left ventricular segments; PSD, peak strain dispersion; SD, standard deviation.

**Table 4:** Detailed comparison of logistic model discriminative performance using the DeLong test.

Composite Model	Single Parameter	Delta AUC	*p*	AIC Composite Model	BIC Composite Model	AIC Single Parameter	BIC Single Parameter
Model 1	MWI dispersion (IQR)	−0.008	0.72101	91.173	96.150	93.421	98.398
Model 1	L-S Work Asymmetry	0.037	0.08841	91.173	96.150	98.635	103.612
Model 2	MWI dispersion (IQR)	0.003	0.91559	89.232	94.210	93.421	98.398
Model 2	L-S Work Asymmetry	0.048	0.07481	89.232	94.210	98.635	103.612
Model 2	PSD	0.080	0.04020	89.232	94.210	108.884	113.862

AIC—Akaike Information Criterion; BIC—Bayesian Information Criterion.

## Data Availability

The original contributions presented in this study are included in the article/Supplementary Material. Further inquiries can be directed to the corresponding author.

## Supplementary Materials

The following supporting information can be downloaded at https://www.mdpi.com/article/10.3390/biomedicines14040941/s1, Table S1: Composite ranking of LV MW indices for model prioritization based on correlation strength with QRS duration (Spearman rho rank) and LBBB discrimination (AUC rank).
