# Supplementary material for: Beyond QRS Duration: Myocardial Work Indices for the Assessment of Left Bundle Branch Block
Source: Biomedicines. 2026 Apr 21;14(4):941. doi: 10.3390/biomedicines14040941 (PMC13113751; doi:10.3390/biomedicines14040941)
Supplement: Supplementary file 1 [file biomedicines-14-00941-s001.zip › biomedicines-4241034-supplementary.pdf]

Table S1. Composite ranking of LV MW indices for model prioritization based on correlation strength with QRS duration (Spearman rho rank) and LBBB discrimination (AUC rank).

Variables	rho	rho rank	AUC	AUC rank	Summary rank
MWI dispersion (IQR) [mmHg%]	0.581	1	0.852	1	2
L-S Work Asymmetry [mmHg%]	0.529	3	0.807	3	6
MWI dispersion (SD) [mmHg%]	0.523	4	0.821	2	6
PSD [ms]	0.545	2	0.775	7	9
MWE dispersion (SD) [mmHg%]	0.511	5	0.802	4	9
MWI dispersion (Range) [mmHg%]	0.469	6	0.800	5	11
MWE dispersion (Range) [mmHg%]	0.450	8	0.780	6	14
GWE [%]	-0.463	7	0.248	8	15
P-AS Work Asymmetry [mmHg%]	0.447	9	0.775	7	16
MWE dispersion (IQR) [mmHg%]	0.441	10	0.709	10	20
GWW [mmHg%]	0.279	11	0.740	9	20
GWI [mmHg%]	-0.245	12	0.456	11	23

Abbreviations: AUC, area under curve; GWE, global work efficiency; GWI, global work index; GWW, global wasted work; IQR, interquartile range; L-S Work Asymmetry, difference in Global Work Index between the basal lateral and basal septal left ventricular segments; MWE, myocardial work efficiency; MWI, myocardial work index; P-AS Work Asymmetry, difference in Global Work Index between the basal posterior and basal anteroseptal left ventricular segments; PSD, peak strain dispersion; SD, standard deviation.
